# Isolation of *Propionibacterium acnes* among the microbiota of primary endodontic infections with and without intraoral communication

**DOI:** 10.1007/s00784-016-1739-x

**Published:** 2016-02-09

**Authors:** Sadia Ambreen Niazi, Hana Suleiman Al Kharusi, Shanon Patel, Kenneth Bruce, David Beighton, Federico Foschi, Francesco Mannocci

**Affiliations:** 1 Department of Restorative Dentistry, King’s College London Dental Institute at Guy’s, King’s and St Thomas’ Hospital, Floor 22, Tower Wing, Guy’s Hospital, St Thomas’ St, London, SE1 9RT UK; 2 Department of Microbiology, King’s College London Dental Institute at Guy’s, King’s and St Thomas’ Hospital, Floor 17, Tower Wing, Guy’s Hospital, St Thomas’ St, London, SE1 9RT UK; 3 Institute of Pharmaceutical Science, King’s College London, Floor 5, 150 Stamford Street, London, SE1 9NH UK; 4 Biomaterials, Biomimetics and Biophotonics Group, King’s College London Dental Institute at Guy’s, King’s and St Thomas’ Hospital, Floor 17, Tower Wing, Guy’s Hospital, St Thomas’ St, London, SE1 9RT UK

**Keywords:** *P. acnes*, Endodontic disease, Nosocomial infection, Fistula

## Abstract

**Objectives:**

The presence of opportunistic pathogens such as *Propionibacterium acnes* (*P. acnes*) may contribute to the endodontic pathology. The presence of *P. acnes* may be influenced by different endodontic conditions.

The aims of the study were firstly, to identify *P. acnes* within the whole cultivable microbiota of primary endodontic infections, to investigate which *P. acnes* phylotypes predominate in such infections and secondly to determine if the presence of an “open” communication (e.g. a sinus) can be associated with the isolation of *P. acnes* from the root canal.

**Material and methods:**

The predominant cultivable microbiota of 15 primary endodontic lesions (7 without communication with the oral environment and 8 with an open communication) were identified using partial 16S ribosomal RNA (rRNA) gene sequence analysis. The identification of the organism was determined by interrogating the Human Oral Microbiome Database. The *P. acnes* isolates were typed on the basis of the *rec*A gene sequence comparison. A neighbor-joining tree was constructed using MEGA 4.1 with the inclusion of known *rec*A sequences.

**Results:**

There was no difference in the number of species identified from lesions without communication (5.86 ± 3.7) and those with communication (5.37 ± 3.6) (*P* > 0.05). PCR-based 16S rRNA gene sequencing revealed *P. acnes* as the most prevalent isolate recovered from lesions with communication. *rec*A gene sequencing revealed two phylogenetic lineages present in lesion with communication, with mainly type I (further split into type IA and type IB) and type II.

**Conclusions:**

The presence of *P. acnes* as opportunistic pathogens has been confirmed and may sustain the traits observed in specific clinical presentations.

**Clinical relevance:**

Clinical management of open lesions may require further disinfection to eliminate opportunistic bacteria.

## Introduction

*Propionibacterium acnes* (*P. acnes*) is a Gram-positive anaerobic/aerotolerant rod that is a resident microbiota of skin, oral cavity, large intestine, conjunctiva and external ear canal [1–4]. Recent studies have identified *P. acnes* as an opportunistic pathogen linked to a wide range of infections and inflammatory conditions including acnes vulgaris [5, 6], sarcoidosis [7], synovitis-acnes-pustulosis-hyperostosis-osteitis syndrome [8, 9] and prostate cancer [10]. The *P. acnes* infections are also linked with trauma and surgery including brain abscesses [11], osteomyelitis after lumbar puncture [12], discitis after surgery [13], spodylodiscitis following epidural catheterization [14], post-operative mediastinitis [15], endophthalmitis [16] and endocarditis [17]. Moreover, foreign body implants are one of the predisposing factors associated with *P. acnes* infections [10, 12, 14].

*P. acnes* have been classified into four highly distinct evolutionary lineages: type IA, IB, II and III, which display differences in inflammatory properties, production of virulence determinants and association with various conditions [18–23]. Type I is usually associated with skin colonization [18], whereas type II and III are almost exclusively associated with infections of implanted prosthesis [19, 24] suggesting their possible role in the pathogenesis of the implant infections. A previous study concluded that *P. acnes* as an opportunistic pathogen was associated much more often than previously reported, with apical periodontitis in teeth with an existing root canal treatment (secondary endodontic infection) [25].

Bacteria may penetrate into the root canal space via caries, trauma and also during the root canal treatment procedure itself due to poor cross infection control and/or inadequate isolation of the operatory field [26]. It has been shown that *P. acnes* type II and III are associated with refractory endodontic infections [25]. It is possible that *P. acnes* may be introduced into the root canal at the time of the root canal obturation or may infect the root canal post-treatment as a result of bacterial leakage at the tooth-restoration interface [25, 27].

The ecology of primary endodontic infection is unique, harboring microbiota, which depend on the presence or absence of a frank communication with the oral cavity. Variation in oxygen tension, availability of metabolites/substrates and replenishment of the biofilm via continuous re-colonization through “open” communications with the oral cavity associated with the sinus tract, full depth periodontal pocket and a history of trauma (resulting in root fracture, tooth avulsion or subluxation) may all significantly alter the microbiota present in primary endodontic infections [26].

The primary aim of the present study was to determine if *P. acnes* is present within the whole cultivable microbiota of primary endodontic infections. The secondary aims were to investigate which specific *recA* phylotypes of *P. acnes* predominate in such infections, and finally to determine if the presence of an “open” communication with the endodontic niche can be associated with a more frequent isolation of *P. acnes* from the root canal space.

## Methods

### Patients selections

Patients recruited into the study received treatment in the Endodontic Department of the Dental Institute at Guys’ and St Thomas’ Hospital, King’s College London. The project was approved by the local ethics committee (South London REC: 05/Q0705/051)*.* The patient had verbal and written information about the purpose of the study, and they gave their written informed consent prior to their inclusion. The selected teeth were single rooted with necrotic pulp (primary lesions). Samples were assigned according to clinical history to group A (absence [Table 1]), or group B (presence [Table 2]) of a communication with the oral environment. In group A, (*n* = 7) samples were collected from the root canal system from teeth which had no macroscopic communication between the tooth and the oral cavity (Table 1). In group B, (*n* = 8) samples were collected from the root canal system from teeth that had evidence of communication with the oral environment either through the presence of a sinus tract, periodontal probing depths of at least 8 mm and/or recent history of trauma resulting in subluxation, luxation and horizontal root fracture in the presence of deep periodontal pocketing (Table 2). All patients were healthy with no active, acute or chronic medical conditions and had not been prescribed antibiotics during the previous month. All significant details about the dental history, clinical and radiographic signs of the involved tooth were recorded (Tables 1 and 2).

**Table 1:** Group A samples without communication

Sample	Sex	Age (Year)	Coronal restoration	Periodontal pockets (mm)	Clinical findings	Tooth	Tx provided
1	M	30	–	2	Necrotic discolored PA lesion	UL1	RCT bleaching
2	F	24	Amalgam	1	Deep restoration, necrotic PA lesion	LR5	RCT PFM crown
3	F	19	Composite	1	Old trauma Necrotic PA lesion	UR2	RCT bleaching
4	M	42	–	3	Enamel infraction necrotic discolored	UR1	RCT bleaching
5	F	27	IRM	2	Deep cavity failed pulp capping	LL3	RCT ceramic crown
6	F	35	Composite	2	Necrotic discolored PA lesion	LR2	RCT Composite core
7	M	20	Composite	1	Deep MOD filling necrotic	UL4	RCT fiber post-composite core
8	M	11	-	2	Old trauma necrotic discolored PA lesion symptomatic	UR1	RCT bleaching

**Table 2:** Group B samples with communication

Sample	Sex	Age (Year)	Coronal restoration	Periodontal pockets (mm)	Clinical findings	Tooth	Tx provided
9	F	21	−	5+	Horizontal root fracture, deep periodontal pocket communicating with the fracture	UR1	RCT MTA Composite build-up
10	F	23	Composite	3	Necrotic abscess sinus symptomatic	UR2	RCT bleaching composite build-up
11	M	15	−	3	Trauma avulsed surgically repositioned and splinted	UR1	RCT bleaching composite build-up
12	M	32	Composite	4	Long standing abscess, sinus for 5 yrs. Symptomatic h/o flare-up mid Tx	UR1	RCT bleaching composite core
13	F	29	−	3	Trauma Lateral luxation Repositioned and splinted	LR2	RCT composite core
14	M	17	−	3	Trauma sub-luxation Repositioned and splinted	UL4	RCT Composite core
15	M	19	−	4	Trauma avulsed surgically repositioned and splinted	LL3	RCT bleaching composite build-up

### Samples collection

After local anesthesia, the tooth to be treated was isolated with the rubber dam. The tooth the clamp and the surrounding dam were cleaned with 30 % (*v*/*v*) hydrogen peroxide and decontaminated with 2.5 % sodium hypochlorite followed by sodium thiosulphate. After decontamination, the isolated tooth and surrounding dam were swabbed to check for contamination. The existing restoration and caries were removed with a sterile bur, after which the access cavity was completed with a new sterile high-speed carbide bur without water-cooling until the pulp chamber was exposed. The content of the root canal space was removed using a combination of sterile K-type or Hedstrom files (Dentsply Maillefer, Baillagues, Switzerland) as well as rotary instruments (ProTaper, Dentsply Maillefer) without any irrigation. All the tissues removed were transferred into 1 ml PRAS medium (Oxyrase, Mansfield, OH, USA). All samples were immediately transported on ice to the laboratory, after which endodontic treatment was completed.

### Microbial analysis of samples

Each sample was dispersed by vortexing with sterile glass beads, diluted in fastidious anaerobe broth (Lab M, UK) and plated onto non-selective media; duplicate plates of fastidious anaerobe agar (FAA) supplemented with 5 % horse blood (Lab M, UK). The FAA plates were incubated anaerobically for 7 days. After incubation, colonies were counted, and predetermined number of colonies (*n* = 30 per patient) were randomly selected by using the grid to divide the plate into different sections for gram-staining and molecular identification.

The swabs taken from the prepared teeth prior to the removal of the restoration were plated directly onto FAA and incubated anaerobically for 7 days.

### Identification of isolates

All randomly selected isolates were subcultured on FAA plates and grown anaerobically for 24 h. Bacterial genomic DNA was extracted by boiling 100 μl of a suspension of the cultured cells prepared in sterile distilled H_2_O for 10 min, followed by cooling on ice for 10 min and centrifuged at 13,000×*g* for 2 min [18]. The supernatant containing the genomic DNA was stored at −20 °C prior to analysis. DNA amplification of a partial fragment of the 16S ribosomal (rRNA) gene of the isolates was performed using universal primers: 9F (5′-3′ GAGTTTGATCCTGGCTCA) and 907R (5′-3′ CGTCAATTCCTTTGAGTT) [28]. The amplification was carried out with the following reaction mixture (final volume, 25 μl): 0.5 μl of 9F forward primers (concentration 10 pmol/μl; MWG, UK), 0.5 μl of 907R reverse primer (concentration 10 pmol/μl; MWG), 23 μl of Reddymix buffer (Thermo Scientific, UK) and 1 μl DNA extract. The thermal cycling conditions included initial denaturation at 94 °C for 10 min, denaturation at 94 °C for 30 s, annealing at 49 °C for 30 s and extension at 72 °C for 90 s, repeated for 34 cycles and final extension at 72 °C for 5 min. The amplified products were run on a 0.5 % agarose gel (Sigma, UK) and visualized under UV transillumination. PCR products were cleaned for the sequence reaction with Microclean (Sigma, UK) according to the manufacturer’s instructions.

Amplicon sequencing was performed by using an ABI Prism BigDye Terminator sequencing kit (Applied Biosystems) with 30 cycles of denaturation at 96 °C for 10 s, annealing at 50 °C for 5 s and extension at 60 °C for 2 min. Sequencing reaction products were run on an ABI 3730xl sequencer Applied Biosystems). All DNA sequences were analyzed, trimmed and aligned using BioEdit software (version 7.0.0; http://www.mbio.ncsu.edu/BioEdit/bioedit.html). The partial gene sequences were identified by a BLAST search of the NCBI database (http://0-www.ncbi.nlm.nih.gov.ilsprod.ilb.neu.edu/BLAST/), the Human Oral Microbiome database (http://www.homd.org/) or the Ribosome Database Project database http://rdp.cme.msu.edu/). Phylogenetic trees were constructed by the neighbor-joining (NJ) method, based on 16S rRNA gene sequence comparisons, using the MEGA (version 4.1) program (http://www.megasoftware.net/).

### Phylotyping of *P. acnes* isolates

Endodontic *P. acnes* isolates identified by partial 16S rRNA gene sequencing were typed by partial *recA* gene sequencing [18]. All selected *P. acnes* isolates were subcultured on FAA and grown for 24 h.

The *P. acnes recA* gene was amplified using primer PAR-1 (positions −96 to −75; 5′-AGCTCGGTGGGGTTCTCTCATC-3′) and primer PAR-2 (positions +1105 to +1083; 5′-GCTTCCTCATACCACTGGTCATC-3′), which generated a 1201-bp amplicon [18]. The reaction mix (final volume, 25 μl) comprised of 0.5 μl of PAR-1 (concentration 10 pmol/μl; Sigma), 0.5 μl of PAR-2 (concentration 10 pmol/μl; Sigma), 23 μl of Reddymix (Thermo Scientific) and 1 μl DNA extract.

The thermal cycling conditions included initial denaturation at 95 °C for 3 min, denaturation at 95 °C for 1 min, annealing at 55 °C for 30 s, and extension at 72 °C for 90 s, repeated for 35 cycles and a final extension at 72 °C for 10 min. Amplified products were run on a 0.5 % agarose gel and visualized under UV transillumination.

Sequencing was performed as described above. The *P. acnes recA* sequences were compared with GenBank sequences AY642055 (type IA), EU687255 (type IB), AY642061 (type II) and DQ672252 (type III). NJ trees were constructed using the Jukes-Cantor method with MEGA (version 4.1) software (www.megasoftware.net/).

### Statistical analysis

Data distributions were compared using χ2 tests; means were compared using the Mann-Whitney *U* test in SPSSPC (Version 21. IBM, USA). Linear regression analysis was used to determine the effect of open or closed oral communication on the presence of *P. acnes*.

## Results

No organisms were recovered from the samples taken from the disinfected tooth surfaces prior to making access into the root canal. The range of organisms cultured from the 15 primary endodontic infections is shown in Tables 1 and 2. From the 15 samples, a mean of 5.6 ± 3.5 taxa were detected. The number of species identified from lesions without communication (5.86 ± 3.7) was not different (*P* > 0.05) than the number from lesions with communication (5.37 ± 3.6).

### Cultivable taxa from primary endodontic infection without “open” communications with the oral cavity

**Fig. 1:**
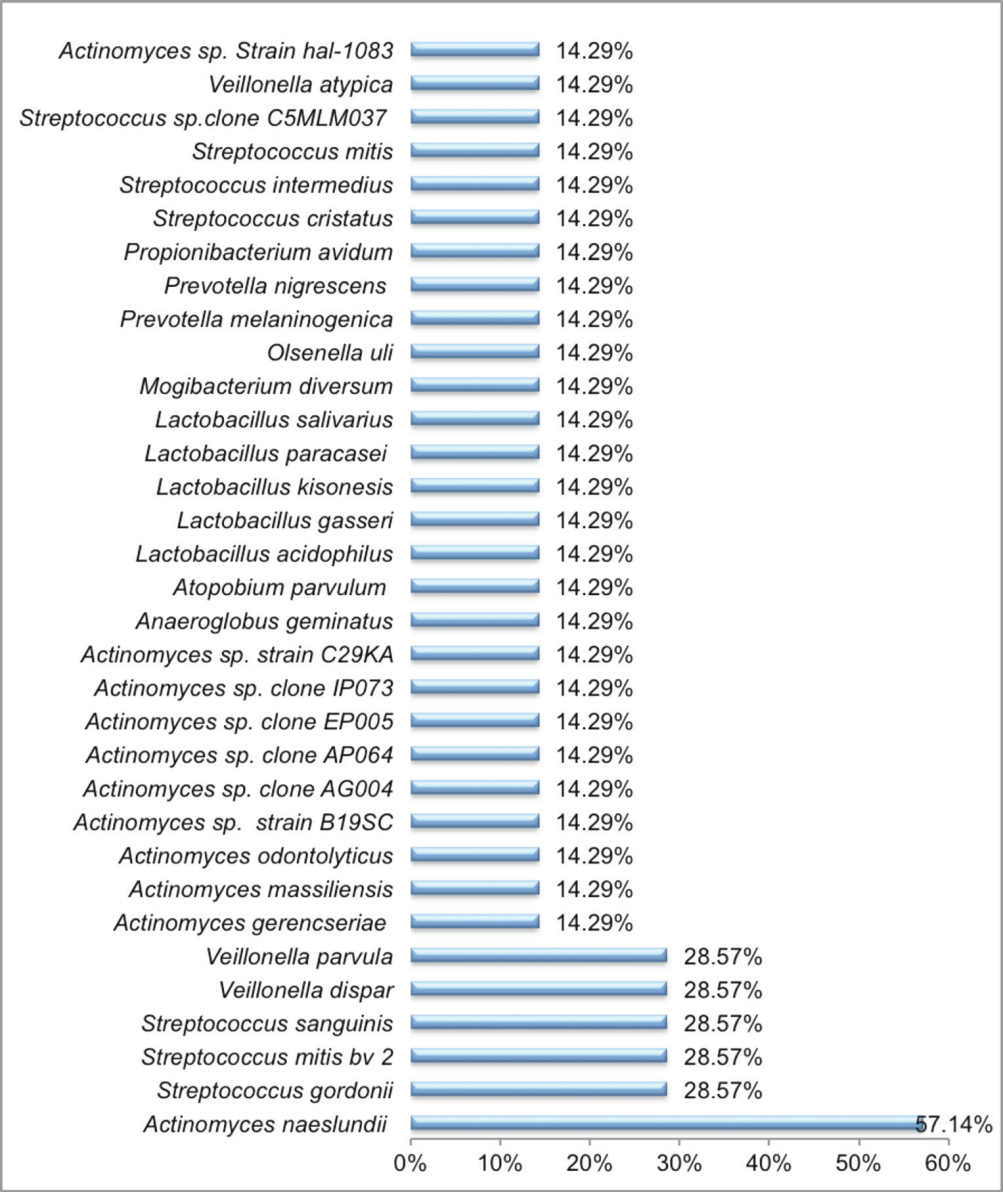
Prevalence of the 33 microbial taxa in 7 primary endodontic lesions without communications with the oral environment

**Fig. 2:**
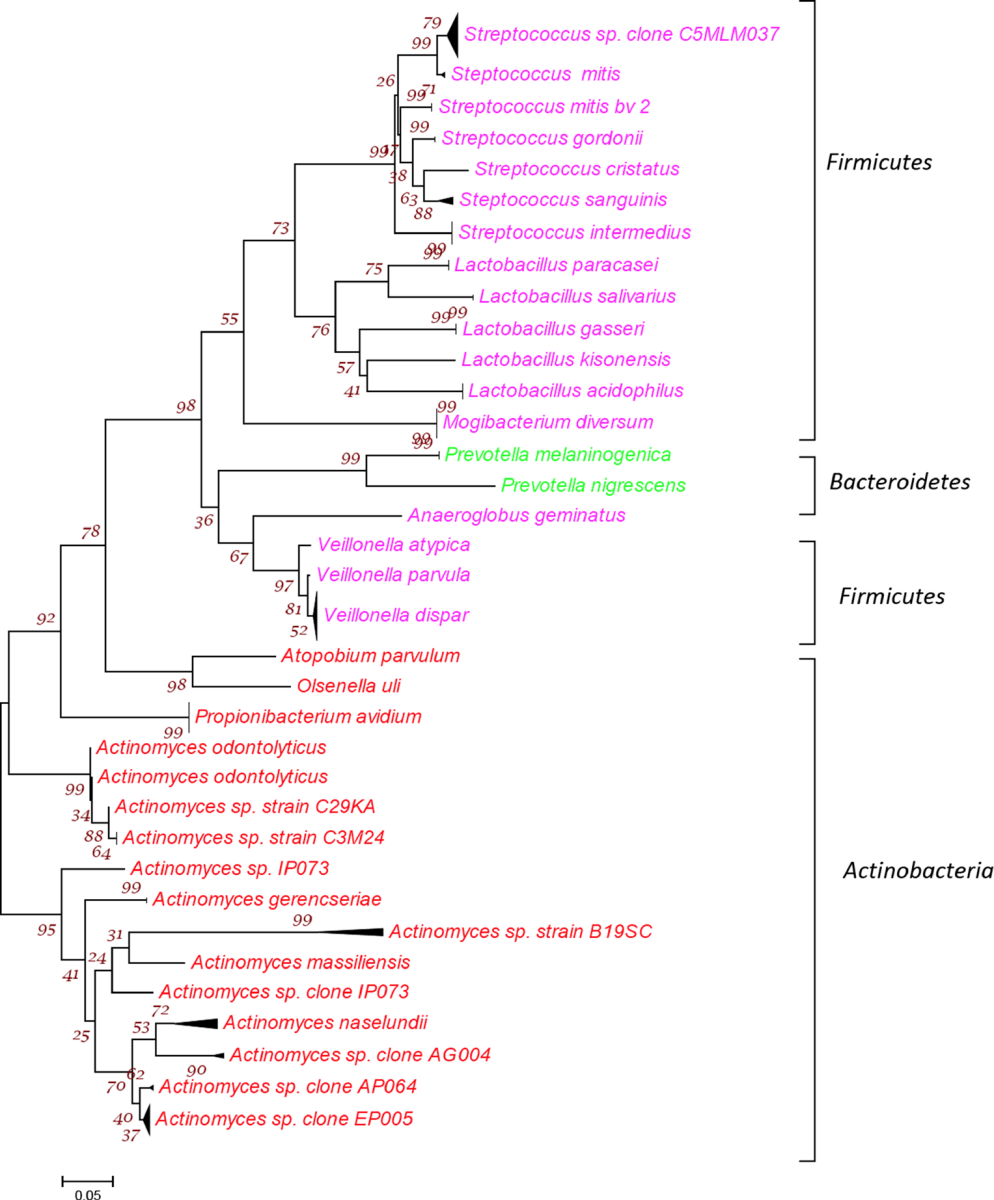
Phylogenetic tree showing all 33 bacterial taxa belonging to 3 phyla from 132 isolates identified from the 7 primary endodontic cases without communications. The tree was constructed by the neighbor-joining method based on 16S rRNA gene sequence comparisons. The *scale bar* represents 0.05 substitutions per nucleotide position. The numbers at the node of the tree indicate bootstrap values for each node out of 500 bootstrap resampling

In the 7 primary endodontic infections without communication with the oral cavity, 33 cultivable bacterial taxa were detected amongst the 132 isolates recovered (Fig. 1). The microbiota of these samples were dominated by the Gram-positive facultative anaerobes, which accounted for 23 of the 31 identified taxa. Furthermore, three Gram-positive obligate anaerobes including *Atopobium parvulum*, *Mogibacterium diversum* and *Olsenella uli* were also identified in these samples*.* Only a minimal number of Gram-negative bacteria were identified in these primary endodontic cases, which comprised obligate anaerobes including two *Prevotella* species (*Prevotella melaninogenica* and *P. nigrescens*) and three species of *Veillonella* (*Veillonella parvula*, *Veillonella dispar* and *Veillonella atypical*) (Fig. 2)*.* No Gram-negative facultative anaerobes were found in these samples. *Actinomyces naeslundii* was the most prevalent bacterial taxa present in 57 % of the cases. This was followed by *Streptococcus gordonii*, *Streptococcus mitis* bv, *Streptococcus sanguinis*, *V. dispar* and *V. parvula* (28.57 % of cases), and all the rest of the taxa were present in 14.29 %.of the cases (Fig. 1).

### Cultivable taxa from primary endodontic infection with “open” communications with the oral cavity

**Fig. 3:**
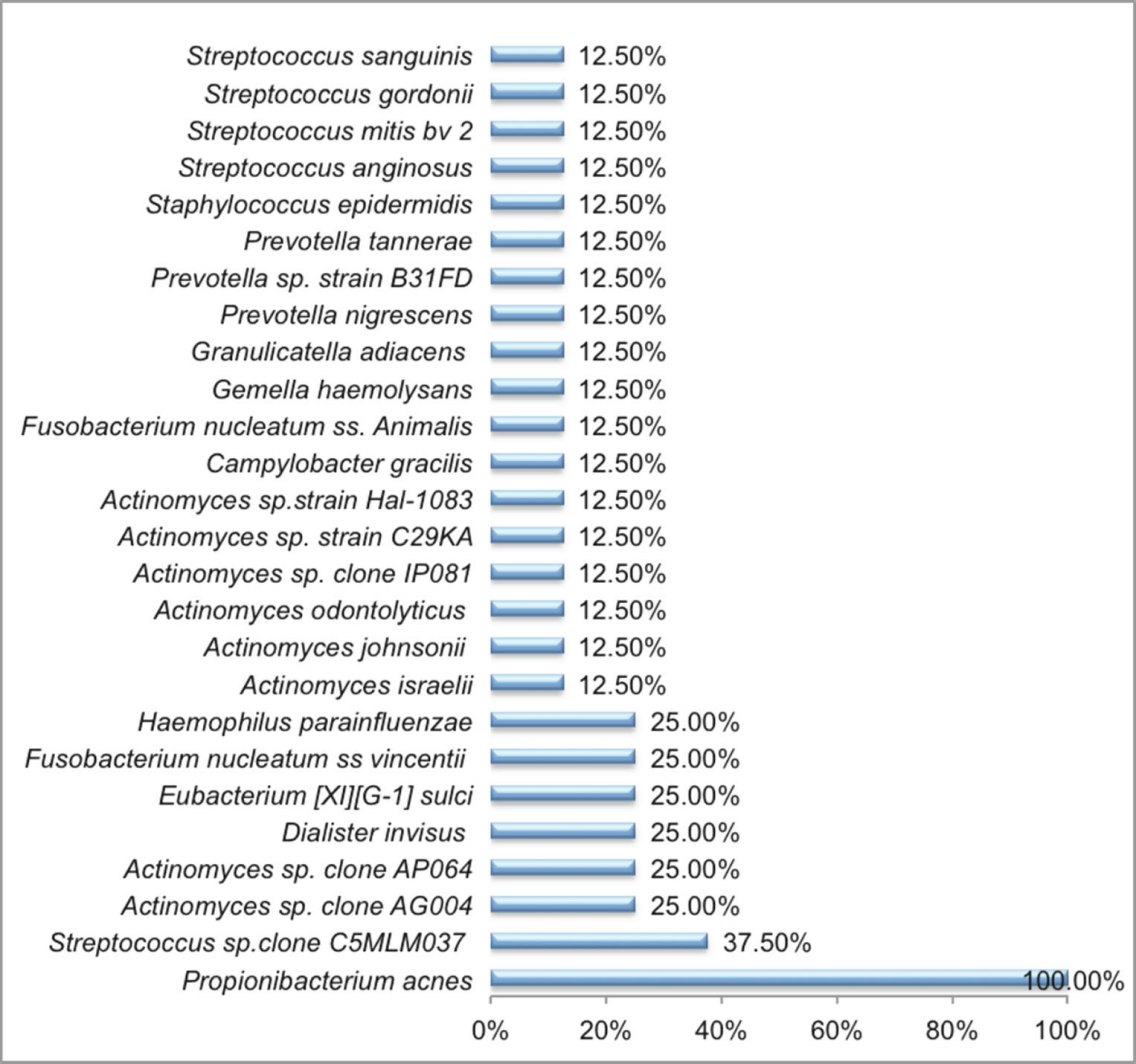
Prevalence of the 26 microbial taxa in 8 primary endodontic lesions with communications with the oral environment

**Fig. 4:**
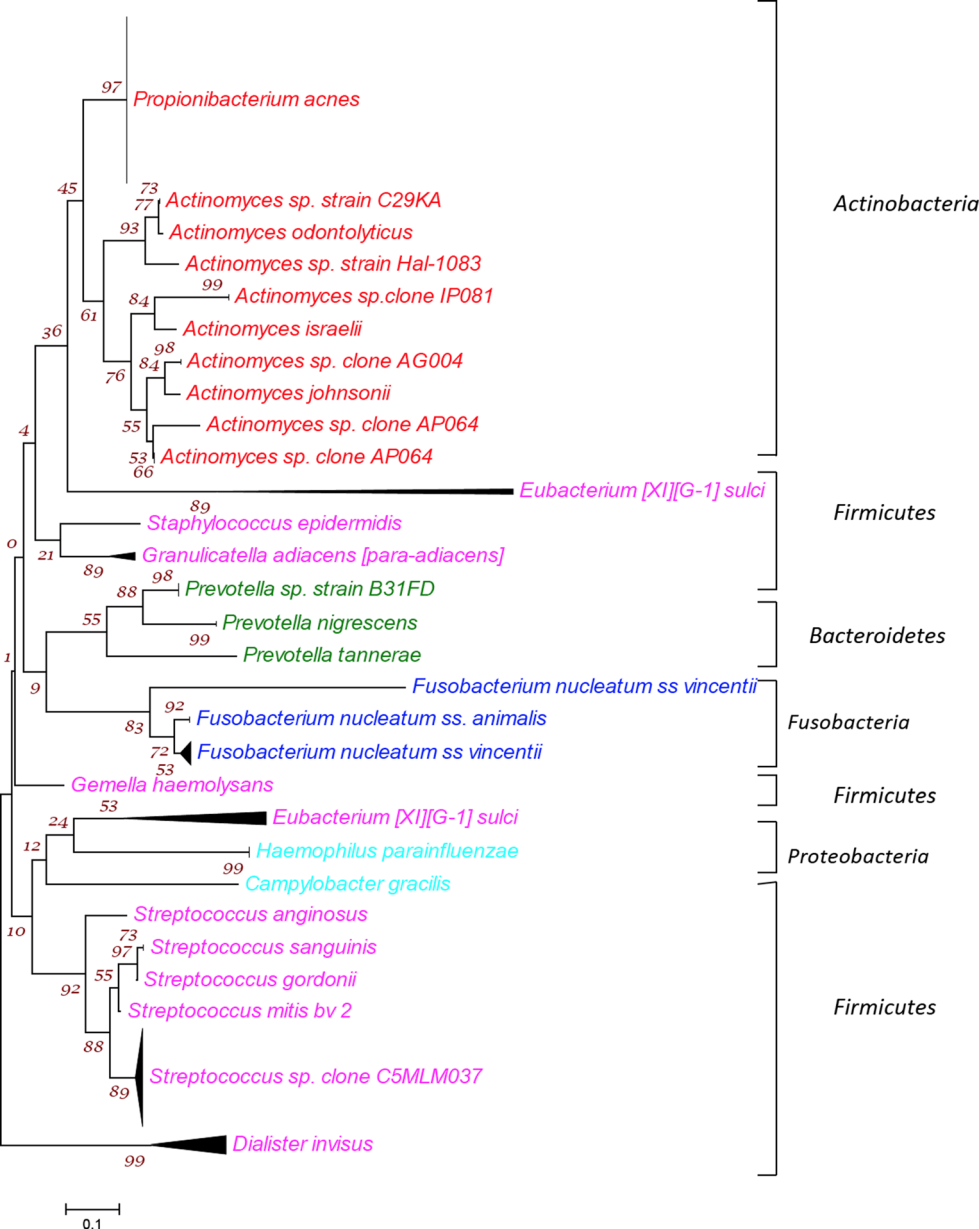
Phylogenetic tree showing all 26 bacterial taxa belonging to 5 phyla from 136 isolates identified from the 8 primary endodontic cases with communications. The tree was constructed by the neighbor-joining method based on 16S rRNA gene sequence comparisons. The *scale bar* represents 0.05 substitutions per nucleotide position. The numbers at the node of the tree indicate bootstrap values for each node out of 500 bootstrap resampling.

From the 8 primary endodontic infections presenting with oral communication, 26 bacterial taxa were detected amongst the 165 isolates recovered (Fig. 3). Microbiota of these infections was mainly dominated by the Gram-positive facultative anaerobes (*n* = 17) with *P. acnes* being the most prevalent bacterial taxa present in all cases (Fig. 3). *Staphylococcus epidermidis* was also recovered. Moreover, these lesions also harbored Gram-positive obligate anaerobes (*n* = 1) including *Eubacterium* [*XI*] [*G-1*] *sulci* and Gram-negative obligate anaerobes (*n* = 6) including one specie of *Dialister* (*Dialister invisus*,), two species of *Fusobacterium* (*Fusobacterium nucleatum ss vincentii* and *Fusobacterium nucleatum ss Animalis*) and three different species of *Prevotella* (*Prevotella tannerae*, *Prevotella nigrescens and Prevotella* sp*. oral strain B31FD*) (Fig. 4).

**Fig. 5:**
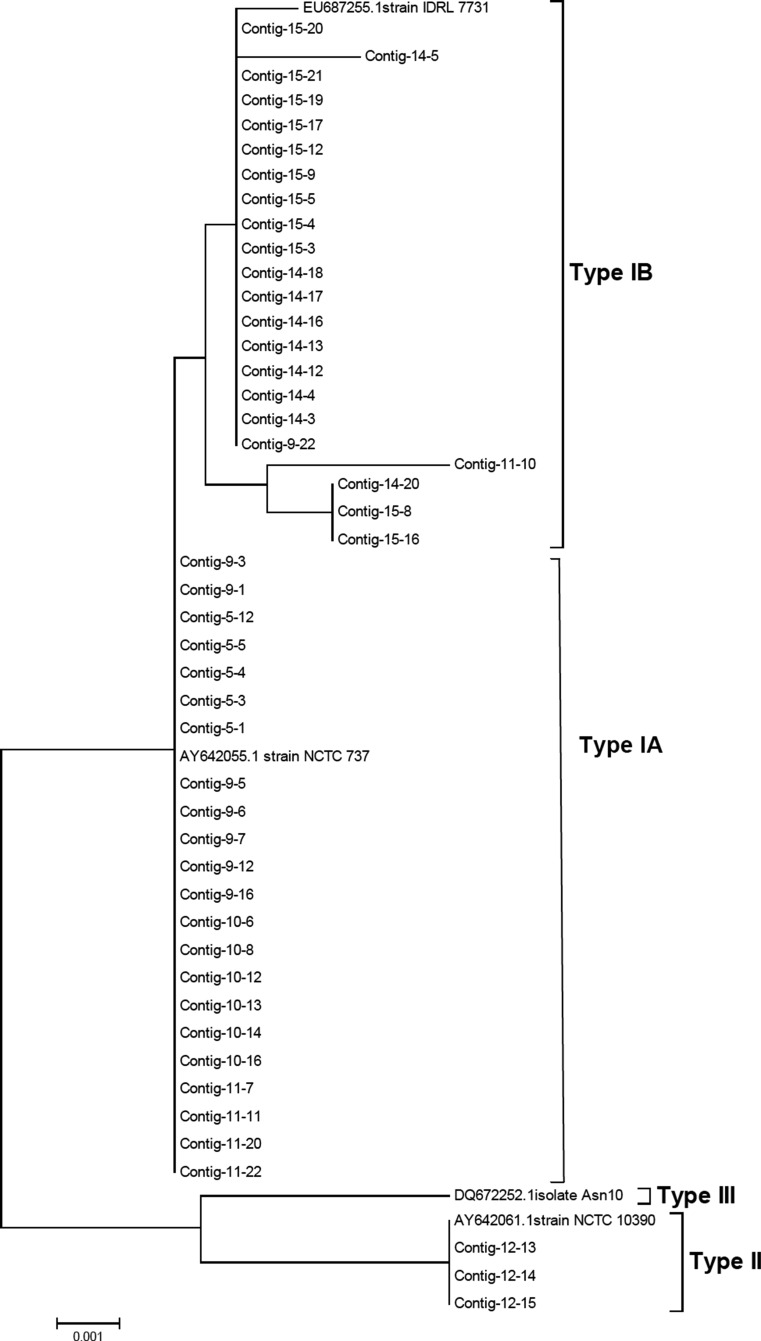
Phylogenetic tree obtained by partial *rec*A sequencing of 47 endodontic *P. acnes* isolates showing three distinct *P. acnes* phylotypes type IA, IB and II. Neighbor-joining (*NJ*) tree was constructed using the Jukes-Cantor method. The *scale bar* represents 0.05 substitutions per nucleotide position. The *numbers at the node of the tree* indicate bootstrap values for each node out of 500 bootstrap resampling

*P. acnes* was recovered from all cases. Moreover, *Streptococcus* sp. *clone C5MLM037* was recovered from 37.50 % cases, followed by *Actinomyces* sp. *clone AG004*, *Actinomyces* sp. *clone AP064*, *D. invisus*, *Eubacterium* [*XI*][*G-1*] *sulci*, *F. nucleatum ss vincentii* and *Haemophilus parainfluenzae* recovered from *25* %*.* All the rest of the taxa were present in 12.50 % cases (Fig. 5).

### Comparison of the richness of bacterial taxa distributed in different phyla in primary endodontic lesions with and without communications with the oral environment

The bacterial taxa identified from the seven primary cases with communication mainly belonged to three phyla including *Firmicutes*, *Actinobacteria* and *Bacteroidetes*. The *Firmicutes* was the phylum with largest number of bacterial taxa that comprised of a variety of seven different species of *Streptococci* and five *Lactobacilli* species, in addition to *Mogibacterium diversum*, *Anaeroglobus geminates* along with three species of *Veillonella.* The next biggest phylum was *Actinobacteria* with 11 different *Actinomyces* species and one *Propionibacteria* including *P. avidum*, *O. uli* and *A. parvulum*. *Bacteroidetes* included two species *of Prevotella* (Fig. 2)*.*

The bacterial taxa identified from the eight primary cases with communication belonged to five different phyla namely *Firmicutes*, *Actinobacteria*, *Bacteroidetes*, *Proteobacteria and Fusobacteria* (Fig. 4). The *Firmicutes* mainly included the Gram-positive anaerobes including five different species of *Streptococci*, *S. epidermidis*, *Granulicatella adiacens*, *Gemella haemolysans* and *Eubacterium* [*XI*][*G-1*] *sulci*, along with one Gram-negative anaerobe including *D. invisus.* The second richest phylum was *Actinobacteria*, which included *P. acnes* and nine different *Actinomyces* species*.* Phylum *Bacteroidetes* comprised of *three species of Prevotella*. Two species of *Fusobacterium* belonging to phylum *Fusobacteria* were also identified in the samples. Moreover, phylum *Proteobacteria* comprised of *Campylobacter gracilis* and *H. parainfluenza* (Fig. 4)*.*

The richness of the bacterial taxa distributed in different phyla identified from primary endodontic lesions with and without communications is shown in Table 3. In *Firmicutes* phylum, the number of representatives is higher in the lesions without communications as compared to the samples with communications. *Proteobacteria* and *Fusobacteria* were exclusively present in samples with communication, with two representatives in each phylum. The distributions were significantly different (*P* < 0.05). Linear regression analysis showed that open oral communication was a significant predictive value for the presence of *P. acnes*.

**Table 3:** Comparison of the bacterial phyla richness in primary endodontic lesions with and without communications with the oral environment

Phyla	Taxa detected in primary endodontic lesions without communication	Taxa detected in primary endodontic lesions with communications
*Firmicutes*	17	10
*Actinobacteria*	14	10
*Bacteriodetes*	2	3
*Proteobacteria*	–	2
*Fusobacteria*	–	2

### Phylotyping of *P. acnes* isolates

Partial *recA* sequences were obtained for 47 endodontic *P. acnes* isolates, and on the basis of sequence alignment, 2 distinct phylogenetic lineages, type I and type II, were observed. Type I was the dominant phylotype, comprising of 44 out of 47 of the endodontic *P. acnes* isolates. The type I isolates segregated into two distinct groups, into which the sequences from known type IA and type IB sequences clustered (Fig. 5).

## Discussion

It is well established that microbial infection of the root canal system is the cause of apical periodontitis [29]. Previous studies by 16S rRNA gene clone libraries demonstrated that 391 bacterial taxa belonging to 82 genera and 9 phyla have been identified in primary endodontic infections [30–33]. We used a culture-based approach to investigate the microbiota of primary endodontic infections with or without communications with the oral environment, along with further molecular identification by specific gene sequencing. With a culture-based approach, fewer taxa may be identified than are detectable with 16S rRNA cloning or cloning combined with culture approaches. In a previous study of five primary endodontic lesions in four patients, a mean of 20.2 taxa were identified using a combined cloning and cultural approach [34], whereas a mean of 6.33 taxa was identified in this study. However, along with cataloging the taxa of these lesions, using culture-based approach we further investigated the phylotyping of the *P. acnes* isolates based on house-keeping gene (*rec*A) sequencing (Table 4 and Table 5).

**Table 4:** Distribution of all 26 bacterial taxa identified among 165 isolates recovered from 8 primary endodontic lesions with communications with oral environment

Organism	Presence in primary endodontic lesions with communication							
Gram-positive organisms	8	9	10	11	12	13	14	15
Obligate anaerobes								
*Eubacterium* [*XI*][*G-1*] *sulci*	−	−	−	−	+	+	−	−
Facultative anaerobes								
*Actinomyces israelii*	+	−	−	−	−	−	−	−
*Actinomyces johnsonii*	+	−	−	−	−	−	−	−
*Actinomyces odontolyticus*	+	−	−	−	−	−	−	−
*Actinomyces sp. clone AG004*	−	+	−	+	−	−	−	−
*Actinomyces sp. clone AP064*	+	−	−	−	−	+	−	−
*Actinomyces sp. clone IP081*	+	−	−	−	−	−	−	−
*Actinomyces sp. strain C29KA*	−	−	−	−	−	−	+	−
*Actinomyces sp. strain Hal-1083*	+	−	−	−	−	−	−	−
*Gemella haemolysans*	−	−	−	−	−	−	+	−
*Granulicatella adiacens*	−	−	−	−	−	−	−	+
*Propionibacterium acnes*	+	+	+	+	+	+	+	+
*Staphylococcus epidermidis*	−	−	−	−	+	−	−	−
*Streptococcus anginosus*	−	−	−	−	−	+	−	−
*Streptococcus gordonii*	+	−	−	−	−	−	−	−
*Streptococcus mitis by 2*	+	−	−	−	−	−	−	−
*Streptococcus sanguinis*	−	−	−	−	−	−	+	−
*Streptococcus* sp*. clone C5MLM037*	−	+	+	+	−	−	−	−
Gram-negative organisms								
Obligate anaerobes								
*Dialister invisus*	−	−	−	−	+	+	−	−
*Fusobacterium nucleatum* ss *vincentii*	−	−	−	−	+	+	−	−
*Fusobacterium nucleatum* ss								
*Animalis*	+	−	−	−	−	−	−	−
*Prevotella nigrescens*	+	−	−	−	−	−	−	−
*Prevotella sp. strain B31FD*	−	−	−	−	+	−	−	−
*Prevotella tannerae*	+	−	−	−	−	−	−	−
Facultative anaerobes								
*Campylobacter gracilis*	+	−	−	−	−	−	−	−
*Haemophilus parainfluenzae*	−	−	−	−	−	−	+	+

**Table 5:** Comparison of the bacterial phyla richness in primary endodontic lesions with and without communications with the oral environment

Phyla	Taxa detected in primary endodontic lesions without communication	Taxa detected in primary endodontic lesions with communications
*Firmicutes*	17	10
*Actinobacteria*	14	10
*Bacteriodetes*	2	3
*Proteobacteria*	–	2
*Fusobacteria*	–	2

There are several avenues for the root canal to become infected. This includes dental caries with involvement of the pulp or as an infection following root canal treatment. It may also arise after a dental trauma. In some instances, there are no obvious cause(s) for root canal infection. In such cases, the crown and the root of the tooth may be intact [35]. All these situations influence the microbiota of endodontic lesions as either dominated by caries organisms or opportunistic bacteria. One of the consequences of trauma may also be vertical root fracture and formation of micro-cracks that may represent a further pathway for open contamination of the root canal space. In the present study, the signs of vertical root fracture were excluded under microscope magnification and in the absence of erratic electronic apex locator reading.

Although all sampled canals had radiographic signs of chronic apical periodontitis indicating a later stage of the disease process, the reported differences in the microbiota between the lesions with and without “open” communication with the oral cavity might be due to their differences in the root canal environment. The exposure of pulp space to the oral environment may allow the direct entry of bacteria, nutrients and oxygen into the root canal space, creating an environment that is radically different from that of root canals with no direct exposure to the oral environment such as those invaded by bacteria associated with caries where a low redox potential and lack of nutrients exist. It was therefore postulated that significant qualitative and quantitative differences in the microbiota might exist between exposed and unexposed canals.

Diverse groups of Gram-positive and Gram-negative bacteria have been identified from the cases included in this study. The taxa found exclusively in cases with communication include *P. acnes*, *S. epidermidis*, *F. nucleatum ss vincentii*, *F. nucleatum ss animalis*, *H. influenza*, *C. gracilis* and *D. invisus*. In cases without communication, a variety of *Lactobacilli* and *Veillonella* were exclusively found along with more species of *Streptococcus* as compared to the ones in cases with communication.

Among the Phyla *Firmicutes*, *D. invisus* was only found in cases with communication. The high prevalence of *D. invisus* was also reported in other studies [36, 37]; their presence is dictated by the ideal environment of an abscess. *Fusobacterium* is also associated with the lesions with abscesses. *F. nucleatum* has been reported to be associated with the more severe form of inter-appointment flare-ups [38]. In this study, there were significant differences between the two cohorts in the prevalence of *F. nucleatum* and *H. parainfluenza* being isolated from approximately 25 % of the communication lesions.

*P. acnes* was the most prevalent bacterial taxa found in all the primary endodontic cases with communication. However, *P. acnes* was not recovered from the cases without communication. This emphasizes the ability of *P. acnes* to gain access through the possible communications into the root canal space and then establish itself as a predominant microbiota of such cases. A previous study showed the presence of *P. acnes* and *S. epidermidis* in the samples from refractory endodontic infections [25]. Other studies that recovered *P. acnes* and *S. epidermidis* considered it as contaminants since they are skin commensals [3, 18, 24, 39, 40]. In a previous study by Niazi et al. (2010), a decontamination protocol was used to remove contaminating organisms from the tooth surface prior to entering the diseased tooth [25]. Similar decontamination protocol was used in this study, thus confirming that *P. acnes* recovered from these primary cases with communication were opportunistic endodontic pathogens rather than contaminants, which is partially confirmed by the lack of *P. acnes* isolated in teeth with no communication with the oral cavity.

*P. acnes* has several virulence factors, in particular protease and polysaccharide formation capabilities are linked to chronic or persistent low-grade implant-associated infections. In these subclinical scenarios, in the absence of positive cultures, this pathogen is probably under-recognized and underestimated [41].

The genotypic analysis of the *P. acnes* isolates using *rec*A gene sequencing showed that type IA and IB were the prevalent phylotypes found in primary cases with communication. These phylotypes probably were derived from patient’s own skin or oral cavity during the exposure and communication of these teeth through the sinus tract, periodontal involvement or root fracture. These might also be the result of a nosocomial infection occurred as a result of manipulation of these teeth during treatment [25, 42]. Type II phylotype of *P. acnes* was only indentified in one case collected from the teeth with a long-standing infections (>5 years) and a history of intermittent flare-ups. Although, the exact source of type II is still not identified, there could be a relationship between the occurrence of these phylotypes and the presence of long-standing infections with “open” communication with the oral cavity. This speculation is also partially supported by the more frequent isolation of philotypes II and III in failed root canal treatment cases (25) which are likely to be associated with longer standing infections.

*P. acnes* is the most prevalent species in primary endodontic infections with a history or clinical evidence of communication with the oral environment, whereas it is absent in lesions without communications. Using *rec*A sequencing, this study showed that *P. acnes* type IA and 1B are associated with primary endodontic infections with communications. *P. acnes* in primary endodontic infections with communications with the oral cavity are likely to act as opportunistic pathogens.

The clinical management of endodontic cases where an open lesion is present may require specific strategies to maximize the chances to eliminate the opportunistic pathogens, such as *P. acnes*. In particular, adopting two stages root canal treatment approach with usage of intermediate dressing may be required even in primary endodontic treatment, where single-stage endodontics was usually considered.
